# A Review of Magnetic Resonance Spectroscopy Studies in Marijuana using Adolescents and Adults

**DOI:** 10.4172/2155-6105.S4-010

**Published:** 2013-04-24

**Authors:** Jennifer T Sneider, Yasmin Mashhoon, Marisa M Silveri

**Affiliations:** 1 Neurodevelopmental Laboratory on Addictions and Mental Health, McLean Imaging Center, McLean Hospital, Belmont, MA, USA; 2 Behavioral Psychopharmacology Research Laboratory, McLean Imaging Center, McLean Hospital, Belmont, MA USA; 3 Department of Psychiatry, Harvard Medical School, Boston, MA, USA

**Keywords:** Marijuana, Cannabis, Neurochemistry, Magnetic resonance spectroscopy, Magnetic resonance spectroscopic imaging, Adolescence, Adulthood

## Abstract

Marijuana (MJ) remains the most widely used illicit drug of abuse, and accordingly, is associated with adverse effects on mental and physical health, and neurocognitive decline. Studies investigating the neurobiology of underlying MJ effects have demonstrated structural and functional alterations in brain areas that contain moderate to high concentrations of cannabinoid (CB1) receptors and that are implicated in MJ-related cognitive decrements. Proton magnetic resonance spectroscopy (^1^H MRS), a non-invasive imaging technique used to assess neurochemistry, has been widely applied to probe a variety of substance-abusing populations. To date, however, there is a relative paucity of MRS published studies characterizing changes in neurometabolite concentrations in MJ users. Thus, the current review provides a summary of data from the eight existing MRS studies of MJ use in adolescents and adults, as well as interpretations and implications of study findings. Future MRS studies that address additional factors such as sex differences, onset and duration of use, abstinence and age, are warranted, and would lead to a more thorough characterization of potential neurochemical correlates of chronic MJ use, which would fill critical gaps in the existing literature.

## Introduction

Marijuana (MJ) research continues to be a major area of investigation, since it remains the most widely used illicit drug of abuse in the United States [1]. Frequent long-term MJ use can have serious adverse effects on mental and physical health, as well as work performance, family and school interactions [2]. According to the 2011 Monitoring the Future study, daily MJ use is on the rise, with approximately one in fifteen high school seniors reporting current daily or near-daily MJ use [3]. It is not surprising that MJ use has been increasing among teens, as perceived risks associated with MJ use have been falling over the past five years, as well as a decline in disapproval of using MJ [3]. The availability of MJ and early age of onset of use has been shown to be highly predictive of MJ use initiation, frequency of use, and MJ dependence [4]. Given that adolescence is a developmental period characterized by rapid functional and structural brain changes, identifying neurodevelopmental vulnerabilities associated with early and escalating MJ use during adolescence and the enduring effects of continued heavy use into adulthood, are critical [5].

Delta-9-tetrahydrocannabinol (Δ9-THC) is the main psychoactive component of MJ, which acts on cannabinoid (CB1) receptors that are densely distributed within brain networks critical for learning, memory, attention, cognitive processing, and motor control [6], Brain regions within these networks that contain moderate to high concentrations of CB1 binding sites include the hippocampus, frontal cortex, inferior temporal gyrus and occipito-temporal gyrus, amygdala, thalamus, cerebellum, and basal ganglia [6–8]. Consistent with the known anatomical distribution of CB1 receptors, studies have shown corresponding MJ-related functional and neurobiological alterations in these brain regions. Accordingly, a number of empirical neuropsychological studies have shown that chronic MJ use negatively impacts attention, frontal executive function, emotional processing, motor skills, and memory, in adolescent [9–11] and adult MJ users [12–16]. While a meta-analytic study failed to report substantial long-term neurocognitive deficits associated with MJ use in adults, notable deficits have been reported in the domains of learning and memory [17]. Impairments in cognitive processing measured across multiple cognitive domains may reflect the consequences of MJ-related neurobiological alterations and/or compensatory reorganization of brain structure and function.

Magnetic resonance imaging (MRI) methods, including diffusion tensor imaging (DTI), have been utilized to investigate the effects of MJ on brain structure [18,21]. Findings have revealed reductions in frontal region fractional anisotropy in long-term MJ users, indicating compromised white matter (WM) fiber bundle integrity, as well as impaired axonal fiber connectivity in the hippocampal fornix and splenium of the corpus callosum, indicating micro structural disturbances to WM fiber trajectories [18,22]. Persistent short-term MJ use during critical neurodevelopmental periods interferes with typical maturation, as adolescent MJ users have shown altered frontal region and insula cortical thickness, reflective of potentially aberrant gray matter (GM) development or maturation [21].

**Figure 1:**
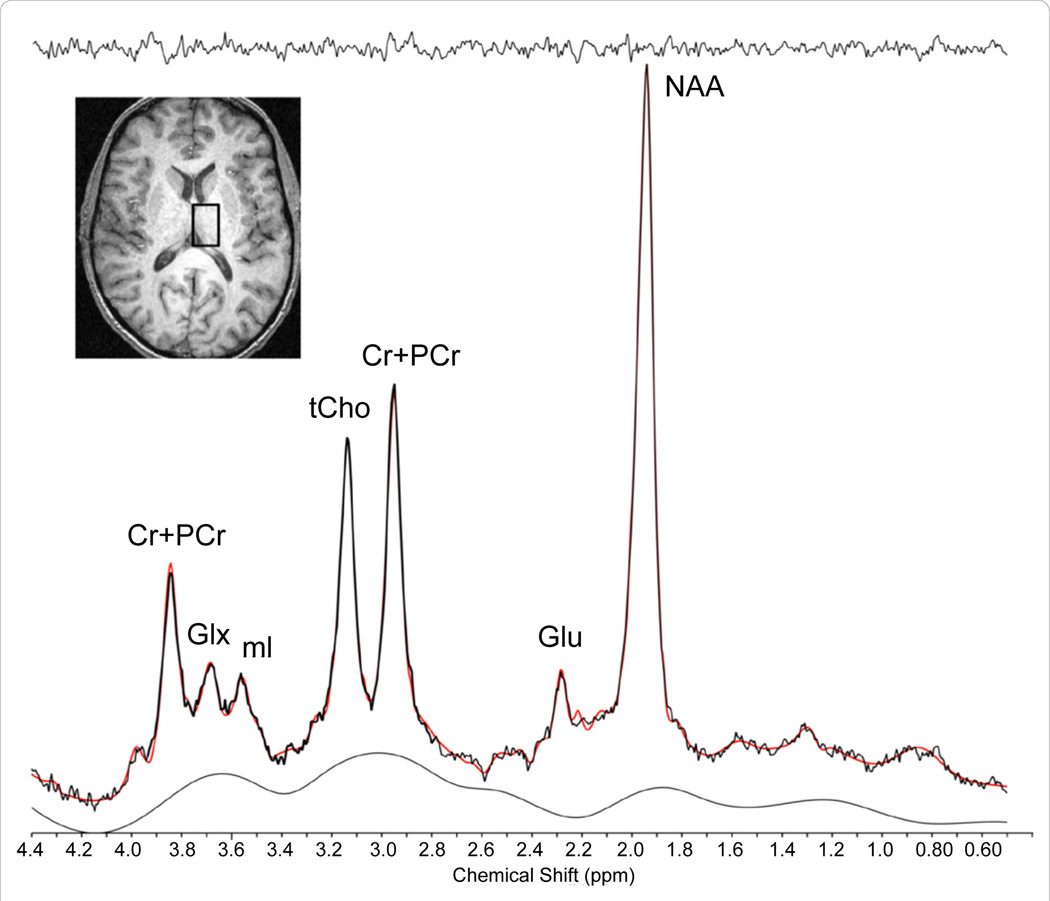
Sample Single Voxel Thalamic ^1^H Spectrum. Axial anatomical image illustrating the placement of a single voxel (2 × 2 × 3cm) in the right thalamus of a healthy subject acquired at 4.0 Tesla using 2D-JPRESS, and the associated ^1^H spectrum (below). Abbreviations: tCr (Cr + PCr), total creatine; Glx, glutamine/glutamine/GABA complex; ml, myo-inositol; tCho, total choline; Glu, glutamate; NAA, N-acetylaspartate.

Functional magnetic resonance imaging (fMRI) has been widely used for studying alterations in functional brain activation related to MJ use during the performance of cognitive challenge tasks [23–27], A recent comprehensive review of fMRI studies is available from Batalla et al., providing a detailed overview of neuroimaging studies of chronic effects of MJ use on brain structure and function [28]. Cerebral blood volume (CBV) studies have also been conducted to investigate MJ effects on cerebral hemodynamics, with dynamic susceptibility contrast magnetic resonance imaging (DSC MRI) demonstrating that while CBV levels begin to normalize with continued abstinence from MJ, specifically in frontal areas, other temporal and cerebellar brain regions show slower CBV decreases after the cessation of active use [29,30],

Proton magnetic resonance spectroscopy (^1^H MRS) is another type of non-invasive imaging technique that has been applied, using single voxel or multiple voxel (magnetic resonance spectroscopic Imaging, MRSI) acquisition schemes, to characterize alterations in neurometabolites that reflect cellular health and bioenergetics [31]. There is a paucity of studies, however, utilizing MRS to investigate the influence of chronic MJ use on brain chemistry [32,33]. As illustrated in Figure 1, *in vivo* ^1^H MRS permits the ability to detect and quantify cerebral metabolites including N-acetyl-aspartate (NAA), myo-inositol (mI), creatine (Cr), cytosolic choline (Cho), and glutamate (Glu). NAA contributes the largest signal, second to unsupressed water, in the proton spectrum, and is found primarily in neurons [34,35]. Though a topic of much debate, NAA has generally been regarded as a biomarker indicating neuronal viability [34]. Total Cr (tCr; creatine plus phosphocreatine) plays a major role in energy metabolism in brain, acting both as an energy buffer by maintaining constant brain adenosine triphosphate (ATP) levels through the creatine kinase reaction and by distributing energy (via mitochondria) within the brain [36,37]. A number of resonances contribute to the Cho signal (i.e., choline-containing compounds including free choline, glycerolphosphocholine and phosphocholine (GPC and PC, respectively), with these compounds being involved in pathways of cellular membrane synthesis and degradation [38]. As a marker of astroglial function, mI actively contributes to cell volume regulation and neuronal energy utilization [39,40]. Finally, Glu is a major excitatory neurotransmitter found in all brain cell types, with the highest concentration generally being found in neurons, or GM tissue.

Characterizing changes in neurometabolite concentrations in areas known to be affected structurally and functionally by chronic MJ use could potentially provide a neurochemical context for these alterations and further substantiate the growing evidence in the field of MJ’s neurobiological and cognitive consequences. Despite the wide application of MRS to study drug effects on brain chemistry, particularly in studies of alcohol abuse and dependence, only eight papers have been published to date that have empirically examined MJ effects on brain chemistry using MRS [41,42]. To this end, the current review focuses on the available MRS studies of MJ use in adults, as well as in adolescents. A general discussion is provided for the overall findings from each study, along with implications and future directions for imaging research on MJ abuse in adolescents and adults.

## Method

Electronic searches were performed using PubMed and Embase to identify published MRS studies using the following key words: ‘marijuana’; ‘cannabis’; in combination with ‘MRS’; ‘MRSI’; or ‘proton MRS’. Methods that utilized other neuroimaging modalities such as computerized tomography (CT), positron emission tomography (PET), structural magnetic resonance imaging (MRI), diffusion tensor imaging (DTI), functional magnetic resonance imaging (fMRI), cerebral blood flow (CBF) or cerebral blood volume (CBV) were not included in the electronic search.

## Results

The available eight studies utilizing MRS to investigate alterations in neurochemistry associated with MJ use are detailed in table 1 and study findings, stratified by adult or adolescent populations, are summarized below.

To date, five MRS studies investigating MJ effects in adult populations have been published. Chang et al. reported lower NAA, ml, Cho, and Glu in the basal ganglia, but elevated thalamic Cr in HIV-negative chronic MJ users [43]. Notably, NAA differences were observed regardless of HIV status, a finding that did not survive post hoc comparisons, and was not observed in the MJ-only group. Metabolite alterations were observed in the absence of neuropsychological performance deficits. In addition, increased frontal WM Glu was associated with history of MJ use in the HIV-positive MJ users, indicating that length of use significantly predicts magnitude of metabolite alterations [43]. In a MRS study by Hermann and colleagues, lower NAA/tCr ratios were observed in the dorsolateral prefrontal cortex (DLPFC) of recreational MJ users [44]. Cowan et al. based on previous evidence that the neuronal marker NAA and the glial marker mI are altered in ecstasy users, examined the influence of lifetime cannabis use in combination with recreational ecstasy use [45]. Lower NAA/Cr levels were evident in Brodmann area (BA) 45 in the frontal lobe, the inferior frontal gyrus region associated with verbal memory processing. The observed metabolite alterations were not observed in BA 18 (secondary visual cortex) or BA 21 (middle temporal gyrus). In a set of studies utilizing MRSI by Silveri et al., evidence for lower mI/ Cr levels were observed in MJ-dependent young men [33,46]. While lower mI/Cr levels were observed on a global level throughout the medial temporal lobe (MTL) region, with greater alterations observed in predominantly WM tissue, additional regional analyses revealed that lower mI/Cr levels were specific to the left thalamus [33,46], Significant correlations were also observed, with mI levels predicting impulsiveness and mood symptoms in the MJ-using group.

To the extent that early initiation of MJ use occurs during the neuromaturational period of adolescence, three MRS studies to date have examined neurochemical alterations associated with MJ use in adolescent populations. In work by Prescot et al., adolescent chronic MJ users demonstrated significantly lower Glu, NAA, tCr and mI in the ACC than healthy age-matched non-users [32]. In addition, recent work by the same group extended MRS alterations in the same set of adolescent MJ users to include lower levels of gamma-aminobutyric acid (GABA), also observed in the ACC [47]. In a study of polydrug using adolescents from South Africa, adolescents who used methamphetamine (MA) + MJ exhibited significantly lower frontal lobe NAA/tCr ratios relative to similarly aged healthy non-users and those who used methamphetamine alone [48],

## Discussion

Emerging empirical data indicate converging evidence for neurochemical alterations in MJ users, with lower NAA being the most frequently observed alteration, apparent in six out of the eight published studies. Lower NAA levels were observed in frontal lobe regions of interest, including the DLPFC, ACC, inferior frontal gyrus and midfrontal GM. Thus, NAA may be a more sensitive marker reflecting MJ neurotoxic effects on neuronal viability. Notably, when frontal lobe regions of interest were included in the MRS study design, NAA alterations were observed in the youngest study participants. The second most common finding was lower mI, observed globally in MTL WM and regionally specific to the left thalamus, in the basal ganglia and in the ACC. Given the role of mI in inflammatory responses, observed lower mI levels are suggestive of MJ-related immunosuppression. Importantly, when age was included as a covariate in the analysis of mI levels in participants who were in their fourth decade of life, mI differences were no longer statistically significant in the basal ganglia, suggesting that age is an important factor when examining interactions between neurochemical levels and MJ use [49]. The one published study documenting evidence of alterations in frontal lobe GABA levels in adolescent MJ users suggests that GABAergic abnormalities may contribute to frontal dysfunctions associated with MJ use, however, it is difficult to discern whether or not this was a focal MJ-related alteration, as no other comparison brain regions were examined [47], Nonetheless, this is consistent with animal models demonstrating that cannabinoids modulate the GABA system via the CB1 receptor with some evidence that interactions between the GABA and cannabinoid systems are more pronounced in adolescent animals likely due in part to ongoing development of the GABA and cannabinoid systems during adolescence [50–56],

Relationships between metabolite levels, clinical and cognitive variables examined in some of these published studies were significant, indicating that metabolite levels may be useful predictors of altered functioning associated with MJ use. The most frequent relationships reported with metabolite levels were with duration or frequency of MJ use: with greater amounts of MJ use being associated with lower Cho, lower NAA and lower mI. Only two published studies, which examined the same cohort of MJ-dependent young men, reported significant correlations between impulsiveness and mood and global mI levels in MTL WM and in the left thalamus. Low mI levels, indicative of suppression of glial function and which may lead to alterations in neuronal function and efficiency, was associated with less impulse control. It is plausible that greater levels of impulsiveness led to the initiation of MJ use in the first place, necessitating the need to characterize antecedents, as well as consequences of MJ use. None of the other MRS studies published to date reported significant relationships between metabolite levels and behavioral data. It is possible that the relatively small sample sizes included in each of these studies, the largest of which included 24 HIV− MJ users, precluded the ability to detect significant differences or relationships due to a lack of statistical power [43]. Clearly brain region of interest and metabolites of interest, along with duration of MJ use, have emerged as important factors for identifying neurochemical correlates of MJ use.

It should also be noted that two of the published studies included MJ-users who were also using additional substances. One study reported findings from MA + MJ adolescent users relative to those using MA alone, thereby limiting conclusions regarding the impact of MJ on neurochemistry, although significant correlations with MJ use were reported [48]. The second study included recreational young adult drug users who were using ecstasy, alcohol, MJ and cocaine, with no MJ-only group or matched non-using comparison group, again limiting conclusions about MJ use alone [45]. Finally, the largest study published to date included MJ users who were both HIV+ and HIV−. Given that the MJ-only group was significantly younger than the other three groups, conclusions drawn from this study are also limited.

There are other methodological factors that should also be considered, including variables associated with drug consumption, co-morbid psychiatric conditions, and age and sex differences. For instance, period of abstinence from MJ use, this was not accounted for in any of the published studies. To the extent that washout period may play an important role in recovery of neurometabolite concentrations, empirical investigation of length of abstinence should be examined in future studies. Structured clinical interviews confirmed MJ dependence in three of the eight studies, whereas the five remaining studies included heavy users. Thus, it is important to consider the potential risk factors and effects of psychiatric comorbidity, especially given recent reports that MJ use is associated with earlier onset of psychosis and psychotic thinking and that lower NAA/tCr ratios in the DLPFC and associated neuropsychological deficits have been reported in patients with schizophrenia [57–60]. The age of the participants in the eight studies ranged from approximately 15 to 45 years, limiting comparisons across studies. However, given that the frontal cortex undergoes the most substantial changes during adolescence, and the onset of drug use often occurs during this period, age of testing in a very important variable to include in MRS investigations of MJ use [61]. Indeed, drug use during the adolescent period may alter the healthy development of the brain via neurotoxic effects of substances on neurochemistry, with polydrug use leading to even greater neurochemical alterations during this critical period of brain development [48]. Finally, there is a significant need for the investigation of sex differences in neurochemistry, underscored by the inclusion of only eight females collectively in MJ-only groups across each of the eight MRS/MRSI studies. In light of sex-specific findings from other non-MRS studies of MJ use, as well as reports on the role of the menstrual cycle phase and sex hormones on neurochemical metabolites identifying potential sex differences in MJ-related alterations of brain metabolite concentrations will significantly contribute to this growing literature [55,62,63],

There are challenges associated with MRS acquisition and quantification that are necessary to consider when interpreting MRS data. Increasing scanner field strength permits increased MR signal, which is field strength dependent, thereby increasing overall sensitivity. Only one published study acquired MRS data at 1.5 Tesla, whereas all other MRS data were acquired at relatively higher field strengths of 3.0 and 4.0 Tesla. The method chosen for spatial localization also can impact the quality of the data collected, with single voxel spectra typically having higher resolution than spectra acquired using MRSI. An advantage of MRSI, however, is the extraction of metabolite spectra from multiple adjacent voxels that can be selected from various regions of interest and investigated during post-processing of MRS data. The choice of spatial localization approach is dependent, therefore, on the a priori hypotheses of the study. It is also important to empirically quantify tissue contributions in the voxel or voxels of interest, as previous studies have shown that proton metabolite concentrations vary with tissue type [64]. Another important factor to consider is the selection of an unbiased reference standard with which to derive absolute metabolite concentrations, rather than assuming that the usual internal references (i.e., Cr, water) are unchanged between populations. As such, the majority of MJ studies conducted to date have utilized either Cr or unsuppressed water as a denominator for determining metabolite ratios, with the exception of the study by Chang and colleagues, who determined absolute concentrations. Cr has typically been considered a relatively stable *in vivo* metabolite peak, which is typically maintained at constant levels in healthy tissue, and therefore routinely used for determining metabolite ratios. The investigation by Chang and colleagues showed that Cr levels varied between subject populations, thereby reducing the likelihood that Cr is an unbiased denominator. The unsuppressed peak arising from water is also routinely used as an internal reference for calculating metabolite ratios, as used by Prescot et al. [32] group, but also is complicated due to the need to discriminate between water in tissue versus water in CSF, which can differ in measurement up to 10–40% [65,66]. Notably, although each of these studies used varying acquisition methods, different field strengths, and examined a variety of regions of interest, there remains converging evidence for an overall trend for MJ-related disruptions in NAA and mI concentrations.

## Conclusion and Future Directions

In summary, this review of the existing MRS/MRSI studies of MJ use demonstrates evidence that smoking MJ alters brain metabolite levels, suggesting a potential neurotoxic effect of marijuana that could be related to a reduction in neuronal viability and altered inflammatory responses associated with chronic use. Future work is warranted to investigate relationships of neurochemical alterations with correlates of clinical and cognitive variables and risk-taking behaviors. Data from this limited collection of studies indicate that this is a significantly understudied area of research, warranting additional investigations, since this review was limited to only eight published studies utilizing MRS to investigate MJ-related alterations in brain chemistry. First, studies should be conducted to characterize the effects of MJ use using a prospective design that follows early onset users from initiation of use, through the critical period of brain development, and into the early 20s, i.e., neurobiological adulthood, when adolescent maturational changes begin to plateau [67,68]. Second, future studies should specifically target MJ use in females, in order to address potential sex-specific differences in the neurochemical consequences of MJ use, which could offer some insight for tailoring individual treatment plans. Third, given that duration of MJ use has been correlated with metabolite alterations across the majority of the available MRS studies, it will be important to characterize and elucidate the effects of extended periods of marijuana exposure, as well as abstinence, on proton metabolite levels and recovery in both younger and older cohorts. Lastly, given that recent pharmacological interventions targeting the GABA system in adults offer some promise for improved recovery from MJ dependence, a more detailed understanding of MJ effects on this inhibitory neurotransmitter system in human subjects is needed [69,70]. Thus, there are a number of future directions for research in this area, utilizing powerful non-invasive methods for assaying *in vivo* neurochemistry, which would fill critical gaps in the existing MJ use literature.

## Figures and Tables

**Table 1:** Proton Magnetic Resonance Spectroscopy Investigations of Marijuana Effects.

Authors & Year	Participants	Age	Marijuana Use Classification	Field Strength & Spatial Localization	Regions of Interest	MRS Results	Clinical or Cognitive MRS Correlates
Chang et al., 2006 [43]	42 HIV+ 21 MJ (3F) 21 CON (4F) 54 HIV− 24 MJ (4F) 30 CON (6F)	42.7 ± 1.7 44.2 ± 3.0 36.3 ± 2.3 42.2 ± 2.2	> 4 days/week	4T Varian SV MRS	Thal, rBG, FWM, CBV, rPar WM, occ WM	↓BG [NAA] HIV+− MJ [ml] [Cho] [Glu] HIV− MJ ↑ Thai [Cr] HIV− MJ ↑ FWM [Glu] HIV+ MJ	↓ BG [Cho] ↑ MJ use ↑ FWM [Glu] ↑ duration use HIV+ MJ
Hermann et al., 2007 [44]	13 MJ (0F) 13 CON (OF)	22.2 ± 2.0 23.0 ± 2.0	near-daily MJ use	1.5T Siemens MRSI	BG, Thal, DLPFC, FWM, HC, VTA, ACC, PCC	↓ DLPFC NAA/tCr MJ	none significant
Cowan et al., 2009 [45]	17 polypro (aF) (ecst, ale, MJ, coc)	21.6 ± 2.7	≥ 500 lifetime episodes of MJ use	3T GE 3T Philips SV MRS	IBA18, BA21.BA45	no non-MJ group comparisons	↑ lifetime MJ use ↓ IBA 45 NAA/Cr
Prescot et al.,2011 [32]	17 MJ (2F) 17 CON (9F)	17.8 ± 1.1 16.2 ± 2.1	≥ 100 past year MJ use	3T Siemens SV MRS	ACC	↓ ACC Glu/H_2_0 NAA/H_2_0 tCr/H_2_0 ml/H_2_0 MJ	none significant
Silveri et al.,2011 [33]	15 MJ-depend (0F) 11 NU (OF)	21.2 ± 3.4 25.0 ± 4.8	DSM-IV criteria for MJ dependence	4T Varian MRSI	multiple voxels in MTL	↓ global ml/Cr MJ ↓ WM ml/Cr MJ	↑ MJ use, ↓ onset use ↓ non-planning ↑ cognitive impulsivity (BIS) ↓ depression (BDI) ↑ tension (POMS)
Sung et al.,2013 [48]	9 MA (6F) 8 MA + MJ (5F) 10 CON (7F)	15.7 ± 1.4 16.2 ± 1.2 16.8 ± 0.6	K-SADS criteria for MJ dependence	3T Siemens SVMRS	midfrontal GM	↓NAA/tCr MA+MJ	↑ lifetime MJ dose ↓ onset MJ use ↓ duration of MJ use
Mashhoon et al., *in press* [46]	13 MJ-depend (0F) 10 NU (0F)	21.3 ± 3.6 24.7 ± 4.9	DSM-IV criteria for MJ dependence	4T Varian MRSI	Thai, TC, POC	↓IThal ml/Cr MJ	↑ cognitive impulsivity
Prescot et al.,2013 [47]	13 MJ (2F) 16 CON (9F)	17.9 ± 1.0 16.0 ± 2.2	≥ 100 past year MJ use	3T Siemens SVMRS	ACC	↓ACC GABA/H_2_0 Glu/H_2_0 NAA/H_2_0 tCr/H_2_0 ml/H_2_0 MJ	none significant

a Number of female participants not indicated

## Abbreviations

^1^H: Proton
ACC: anterior cingulate cortex
BA: Brodmann Area
BDI: Beck Depression Inventory
BG: basal ganglia
BIS: Barratt Impulsiveness Scale
CBV: Cerebral Blood Volume
Cho: choline
CON: healthy control group
Cr: creatine
CSF: Cerebrospinal fluid
DLPFC: dorsolateral prefrontal cortex
DSC: Dynamic Susceptibility Contrast
DTI: Diffusion Tensor Imaging
F: female
fMRI: Functional magnetic resonance imaging
FWM: frontal white matter
GABA: gamma-aminobutyric acid
Gin: Glutamine
Glu: glutamate
Glx: Glutamine/Glutamate/GABA complex
GM: gray matter
H20: normalized water signal
HC: hippocampus
K-SADS: Kiddie-Schedule for Affective Disorders and Schizophrenia
l: left
MA: methamphetamine
mI: myo-Inositol
MJ: marijuana
MRI: Magnetic Resonance Imaging
MRS: magnetic resonance spectroscopy
MRSI: magnetic resonance spectroscopic imaging
MTL: medial temporal lobe
NAA: N-acetyl-aspartate
NU: non-using
Occ: occipital
Par: parietal
PCC: posterior cingulate cortex
PCr: phosphocreatine
POC: parieto-occipital cortex
POMS: Profile of Mood States
r: right
rec: recreational
SV: single voxel
t: total
T: Tesla
TC: temporal cortex
Thai: thalamus
THC: Δ9 tetrahydrocannabinol
VTA: ventral tegmental area
WM: white matter

## References

[1] NIDA (2009). National Institute on Drug Abuse. NIDA InfoFacts.

[2] TEDS (2012). Substance Abuse and Mental Health Services Administration (Center for Behavioral Health Statistics and Quality).

[3] Johnston LD, O’Malley PM, Bachman JG, Schulenberg JE (2012). Monitoring the Future national results on adolescent drug use Overview of key findings 2011. Institute for Social Research, The University of Michigan Ann Arbor.

[4] von Sydow K, Lieb R, Pfister H, Höfler M, Wittchen HU (2002). What predicts incident use of cannabis and progression to abuse and dependence? A 4-year prospective examination of risk factors in a community sample of adolescents and young adults. Drug Alcohol Depend.

[5] Paus T (2005). Mapping brain maturation and cognitive development during adolescence. Trends Cogn Sci.

[6] Glass M, Dragunow M, Faull RL (1997). Cannabinoid receptors in the human brain: a detailed anatomical and quantitative autoradiographic study in the fetal, neonatal and adult human brain. Neuroscience.

[7] Herkenham M, Lynn AB, Johnson MR, Melvin LS, de Costa BR (1991). Characterization and localization of cannabinoid receptors in rat brain: a quantitative in vitro autoradiographic study. J Neurosci.

[8] Terry GE, Hirvonen J, Liow JS, Zoghbi SS, Gladding R (2010). Imaging and quantitation of cannabinoid CB1 receptors in human and monkey brains using (18)F-labeled inverse agonist radioligands. J Nucl Med.

[9] Gonzalez R, Swanson JM (2012). Long-term effects of adolescent-onset and persistent use of cannabis. Proc Natl Acad Sci U S A.

[10] Meier MH, Caspi A, Ambler A, Harrington H, Houts R (2012). Persistent cannabis users show neuropsychological decline from childhood to midlife. Proc Natl Acad Sci U S A.

[11] Solowij N, Jones KA, Rozman ME, Davis SM, Ciarrochi J (2011). Verbal learning and memory in adolescent cannabis users, alcohol users and non-users. Psychopharmacology (Berl).

[12] Battisti RA, Roodenrys S, Johnstone SJ, Respondek C, Hermens DF (2010). Chronic use of cannabis and poor neural efficiency in verbal memory ability. Psychopharmacology (Berl).

[13] Pope HG, Gruber AJ, Hudson JI, Huestis MA, Yurgelun-Todd D (2001). Neuropsychological performance in long-term cannabis users. Arch Gen Psychiatry.

[14] Pope HG, Yurgelun-Todd D (1996). The residual cognitive effects of heavy marijuana use in college students. JAMA.

[15] Whitlow CT, Liguori A, Livengood LB, Hart SL, Mussat-Whitlow BJ (2004). Long-term heavy marijuana users make costly decisions on a gambling task. Drug Alcohol Depend.

[16] Crean RD, Crane NA, Mason BJ (2011). An evidence based review of acute and long-term effects of cannabis use on executive cognitive functions. J Addict Med.

[17] Grant I, Gonzalez R, Carey CL, Natarajan L, Wolfson T (2003). Non-acute (residual) neurocognitive effects of cannabis use: a meta-analytic study. J Int Neuropsychol Soc.

[18] Gruber SA, Silveri MM, Dahlgren MK, Yurgelun-Todd D (2011). Why so impulsive? White matter alterations are associated with impulsivity in chronic marijuana smokers. Exp Clin Psychopharmacol.

[19] Gruber SA, Yurgelun-Todd DA (2005). Neuroimaging of marijuana smokers during inhibitory processing: a pilot investigation. Brain Res Cogn Brain Res.

[20] Cousijn J, Wiers RW, Ridderinkhof KR, van den Brink W, Veltman DJ (2012). Grey matter alterations associated with cannabis use: results of a VBM study in heavy cannabis users and healthy controls. Neuroimage.

[21] Lopez-Larson MP, Bogorodzki P, Rogowska J, McGlade E, King JB (2011). Altered prefrontal and insular cortical thickness in adolescent marijuana users. Behav Brain Res.

[22] Zalesky A, Solowij N, Yücel M, Lubman DI, Takagi M (2012). Effect of long-term cannabis use on axonal fibre connectivity. Brain.

[23] Gruber SA, Rogowska J, Yurgelun-Todd DA (2009). Altered affective response in marijuana smokers: an FMRI study. Drug Alcohol Depend.

[24] Sneider JT, Gruber SA, Rogowska J, Silveri MM, Yurgelun-Todd DA (in press). A preliminary study of functional brain activation among marijuana users during performance of a virtual water maze task. J Addiction.

[25] Hester R, Nestor L, Caravan H (2009). Impaired error awareness and anterior cingulate cortex hypoactivity in chronic cannabis users. Neuropsychopharmacology.

[26] Becker B, Wagner D, Gouzoulis-Mayfrank E, Spuentrup E, Daumann J (2010). The impact of early-onset cannabis use on functional brain correlates of working memory. Prog Neuropsychopharmacol Biol Psychiatry.

[27] Wesley MJ, Hanlon CA, Porrino LJ (2011). Poor decision-making by chronic marijuana users is associated with decreased functional responsiveness to negative consequences. Psychiatry Res.

[28] Batalla A, Bhattacharyya S, Yücel M, Fusar-Poli P, Crippa JA (2013). Structural and functional imaging studies in chronic cannabis users: a systematic review of adolescent and adult findings. PLoS One.

[29] Sneider JT, Pope HG, Silveri MM, Simpson NS, Gruber SA (2006). Altered regional blood volume in chronic cannabis smokers. Exp Clin Psychopharmacol.

[30] Sneider JT, Pope HG, Silveri MM, Simpson NS, Gruber SA (2008). Differences in regional blood volume during a 28-day period of abstinence in chronic cannabis smokers. Eur Neuropsychopharmacol.

[31] Bittsansky M, Vybohova D, Dobrota D (2012). Proton magnetic resonance spectroscopy and its diagnostically important metabolites in the brain. Gen Physiol Biophys.

[32] Prescot AP, Locatelli AE, Renshaw PF, Yurgelun-Todd DA (2011). Neurochemical alterations in adolescent chronic marijuana smokers: a proton MRS study. Neuroimage.

[33] Silveri MM, Jensen JE, Rosso IM, Sneider JT, Yurgelun-Todd DA (2011). Preliminary evidence for white matter metabolite differences in marijuana-dependent young men using 2D J-resolved magnetic resonance spectroscopic imaging at 4 Tesla. Psychiatry Res.

[34] Moffett JR, Ross B, Arun P, Madhavarao CN, Namboodiri AM (2007). N-Acetylaspartate in the CNS: from neurodiagnostics to neurobiology. Prog Neurobiol.

[35] Tsai G, Coyle JT (1995). N-acetylaspartate in neuropsychiatric disorders. Prog Neurobiol.

[36] Andres RH, Ducray AD, Schlattner U, Wallimann T, Widmer HR (2008). Functions and effects of creatine in the central nervous system. Brain Res Bull.

[37] Wyss M, Kaddurah-Daouk R (2000). Creatine and creatinine metabolism. Physiol Rev.

[38] Bayindir Y, Firat AK, Kayabas U, Alkan A, Yetkin F (2012). Increased membrane turnover in the brain in cutaneous anthrax without central nervous system disorder: a magnetic resonance spectroscopy study. Med Hypotheses.

[39] Coupland NJ, Ogilvie CJ, Hegadoren KM, Seres P, Hanstock CC (2005). Decreased prefrontal Myo-inositol in major depressive disorder. Biol Psychiatry.

[40] Maragakis NJ, Rothstein JD (2006). Mechanisms of Disease: astrocytes in neurodegenerative disease. Nat Clin Pract Neurol.

[41] Licata SC, Renshaw PF (2010). Neurochemistry of drug action: insights from proton magnetic resonance spectroscopic imaging and their relevance to addiction. Ann N Y Acad Sci.

[42] Meyerhoff DJ, Durazzo TC, Ende G (2013). Chronic alcohol consumption, abstinence and relapse: brain proton magnetic resonance spectroscopy studies in animals and humans. Curr Top Behav Neurosci.

[43] Chang L, Cloak C, Yakupov R, Ernst T (2006). Combined and independent effects of chronic marijuana use and HIV on brain metabolites. J Neuroimmune Pharmacol.

[44] Hermann D, Sartorius A, Welzel H, Walter S, Skopp G (2007). Dorsolateral prefrontal cortex N-acetylaspartate/total creatine (NAA/tCr) loss in male recreational cannabis users. Biol Psychiatry.

[45] Cowan RL, Joers JM, Dietrich MS (2009). N-acetylaspartate (NAA) correlates inversely with cannabis use in a frontal language processing region of neocortex in MDMA (Ecstasy) poly drug users a 3 T magnetic resonance spectroscopy study. Pharmacol Biochem Behav.

[46] Mashhoon Y, Jensen JE, Sneider JT, Yurgelun-Todd D, Silveri MM (in press). Lower left thalamic myo-inositol levels associated with greater cognitive impulsivity in marijuana-dependent young men preliminary spectroscopic evidence at 4T. J Addict Res Ther.

[47] Prescot AP, Renshaw PF, Yurgelun-Todd DA (2013). γ-Amino butyric acid and glutamate abnormalities in adolescent chronic marijuana smokers. Drug Alcohol Depend.

[48] Sung YH, Carey PD, Stein DJ, Ferrett HL, Spottiswoode BS (2013). Decreased frontal N-acetylaspartate levels in adolescents concurrently using both methamphetamine and marijuana. Behav Brain Res.

[49] Cohen-Gilbert JE, Jensen JE, Silveri MM Adolescent cognitive and neurobiological development Understanding the contribution of neurochemistry. Dev Psychopathol.

[50] Pistis M, Ferraro L, Pira L, Flore G, Tanganelli S (2002). Delta(9)-tetrahydrocannabinol decreases extracellular GABA and increases extracellular glutamate and dopamine levels in the rat prefrontal cortex an in vivo microdialysis study. Brain Res.

[51] Katona I, Sperlágh B, Sík A, Käfalvi A, Vizi ES (1999). Presynaptically located CB1 cannabinoid receptors regulate GABA release from axon terminals of specific hippocampal interneurons. J Neurosci.

[52] Ferraro L, Tomasini MC, Cassano T, Bebe BW, Siniscalchi A (2001). Cannabinoid receptor agonist WIN 55,212-2 inhibits rat cortical dialysate gamma-aminobutyric acid levels. J Neurosci Res.

[53] Kang-Park MH, Wilson WA, Kuhn CM, Moore SD, Swartzwelder HS (2007). Differential sensitivity of GABA A receptor-mediated IPSCs to cannabinoids in hippocampal slices from adolescent and adult rats. J Neurophysiol.

[54] Coyle JT, Enna SJ (1976). Neurochemical aspects of the ontogenesis of GABAnergic neurons in the rat brain. Brain Res.

[55] Silveri MM, Sneider JT, Crowley DJ, Covell MJ, Acharya D (2013). Frontal Lobe Î³-Aminobutyric Acid Levels During Adolescence: Associations with Impulsivity and Response Inhibition. Biol Psychiatry.

[56] Belue RC, Howlett AC, Westlake TM, Hutchings DE (1995). The ontogeny of cannabinoid receptors in the brain of postnatal and aging rats. Neurotoxicol Teratol.

[57] Bossong MG, Niesink RJ (2010). Adolescent brain maturation, the endogenous cannabinoid system and the neurobiology of cannabis-induced schizophrenia. Prog Neurobiol.

[58] Large M, Sharma S, Compton MT, Slade T, Nielssen O (2011). Cannabis use and earlier onset of psychosis: a systematic metaanalysis. Arch Gen Psychiatry.

[59] González-Pinto A, Vega P, Ibáñnez B, Mosquera F, Barbeito S (2008). Impact of cannabis and other drugs on age at onset of psychosis. J Clin Psychiatry.

[60] Molina V, Sánchez J, Reig S, Sanz J, Benito C (2005). N-acetyl-aspartate levels in the dorsolateral prefrontal cortex in the early years of schizophrenia are inversely related to disease duration. Schizophr Res.

[61] Spear LP (2000). The adolescent brain and age-related behavioral manifestations. Neurosci Biobehav Rev.

[62] Epperson CN, O’Malley S, Czarkowski KA, Gueorguieva R, Jatlow P (2005). Sex, GABA, and nicotine: the impact of smoking on cortical GABA levels across the menstrual cycle as measured with proton magnetic resonance spectroscopy. Biol Psychiatry.

[63] Schepis TS, Desai RA, Cavallo DA, Smith AE, McFetridge A (2011). Gender differences in adolescent marijuana use and associated psychosocial characteristics. J Addict Med.

[64] Maudsley AA, Domenig C, Govind V, Darkazanli A, Studholme C (2009). Mapping of brain metabolite distributions by volumetric proton MR spectroscopic imaging (MRSI). Magn Reson Med.

[65] Gasparovic C, Song T, Devier D, Bockholt HJ, Caprihan A (2006). Use of tissue water as a concentration reference for proton spectroscopic imaging. Magn Reson Med.

[66] Mlynárik V, Kohler I, Gambarota G, Vaslin A, Clarke PG (2008). Quantitative proton spectroscopic imaging of the neurochemical profile in rat brain with microliter resolution at ultra-short echo times. Magn Reson Med.

[67] Gogtay N, Giedd JN, Lusk L, Hayashi KM, Greenstein D (2004). Dynamic mapping of human cortical development during childhood through early adulthood. Proc Natl Acad Sci U S A.

[68] Sowell ER, Thompson PM, Toga AW (2004). Mapping changes in the human cortex throughout the span of life. Neuroscientist.

[69] Lile JA, Kelly TH, Hays LR (2012). Separate and combined effects of the GABA reuptake inhibitor tiagabine and Î”9-THC in humans discriminating Δ9-THC. Drug Alcohol Depend.

[70] Mason BJ, Crean R, Goodell V, Light JM, Quello S (2012). A proof-of-concept randomized controlled study of gabapentin: effects on cannabis use, withdrawal and executive function deficits in cannabis-dependent adults. Neuropsychopharmacology.

